# HPV status impacts oncobacteria abundance and prognostic relevance in head and neck squamous cell carcinoma

**DOI:** 10.1038/s41388-025-03463-4

**Published:** 2025-06-10

**Authors:** Travis D. Kerr, Natalie L. Silver, Radhika Duggal, Jin Dai, Hannah Simmons, Subha Singh, Akeesha A. Shah, Kristianna M. Fredenburg, Apollo R. Stacy, Daniel J. McGrail

**Affiliations:** 1 Center for Immunotherapy and Precision Immuno-Oncology, Lerner Research Institute, Cleveland Clinic, Cleveland, OH USA; 2 Department of Biochemistry and Molecular Biology, University of Miami, Miller School of Medicine, Miami, FL USA; 3 Head and Neck Institute, Cleveland Clinic, Cleveland, OH USA; 4 Department of Molecular Medicine, Cleveland Clinic Lerner College of Medicine, Case Western Reserve University, Cleveland, OH USA; 5 Department of Pathology and Laboratory Medicine, Cleveland Clinic, Cleveland, OH USA; 6 Department of Pathology, University of Florida College of Medicine, Gainesville, FL USA; 7 Department of Cardiovascular and Metabolic Sciences, Lerner Research Institute, Cleveland Clinic, Cleveland, OH USA

**Keywords:** Head and neck cancer, Cancer microenvironment, Prognostic markers

## Abstract

The intratumoral microbiome is emerging as an intrinsic microenvironment feature of some cancers, most notably those along the digestive tract. Opportunistic pathogenic bacteria, such as *Fusobacterium nucleatum*, can enrich within certain tumors, ultimately leading to alterations in the tumor microenvironment. However, why some tumors have a higher abundance of tumor-associated bacteria, or oncobacteria, than others remains unknown. To address this question, we quantified the presence of oncobacteria in head and neck squamous cell carcinomas (HNSCCs). We found that accumulation of oncobacteria was independent of tumor stage and size, as well as patient characteristics. In contrast, we discovered that human papillomavirus (HPV)-negative tumors exhibited significantly higher accumulation of oncobacteria than HPV+ tumors. Furthermore, the abundance of oncobacteria was associated with worse overall survival in HPV+ tumors. These findings were validated in an independent cohort. Subsequent analysis of Epstein-Barr virus (EBV)+ gastric cancer suggests this phenomenon generalizes to other virally mediated cancers. Co-culture studies of HNSCC cell lines with *Fusobacterium nucleatum* demonstrated that HPV-negative cells have enhanced proliferation in the presence of *Fusobacterium nucleatum* compared to HPV+ cells, suggestive of tumor cell intrinsic determinants of oncobacteria accumulation. Together, these results illuminate tumor features that contribute to the accumulation of oncobacteria.

## Introduction

Head and neck squamous cell carcinoma (HNSCC) is the eighth most common cancer globally [1, 2]. Genetic and environmental factors both contribute to the development of HNSCC, with males being more susceptible than females and lifestyle factors such as alcohol consumption and tobacco use also increasing risk. In recent years, there has been a rise in human papillomavirus (HPV)-associated oropharyngeal squamous cell carcinoma (OPSCC), which is responsible for the overall increase in HNSCC incidence in the United States [3–9]. In fact, the recent increase in cases of HPV-associated OPSCC has surpassed that of HPV-related cervical cancers and is projected to continue to increase over the next 30–40 years [10]. Treatment for advanced HNSCC requires aggressive therapy, including surgery and/or chemo/radiation, which can result in decreased quality of life associated with disfigurement and functional impairments to swallowing, speech, and taste [11, 12]. Furthermore, response to these first-line treatments can be poor in the recurrent/metastatic setting, highlighting an unmet need to understand factors influencing treatment resistance and disease progression. Additionally, only a minority of HNSCC patients (approximately 20%) respond to immune checkpoint blockade therapy, leaving non-responders with few remaining therapeutic options [13].

The human oral microbiome consists of more than seven hundred species of bacteria, over one hundred species of fungi, and even some species of protozoa [14–16]. The oral microbiome exhibits the second highest level of alpha diversity, following only the gut microbiome, and plays a key role in the maintenance of a normal physiologic environment [14, 15, 17]. Disruption to microbiome diversity, or dysbiosis, has been shown to be associated with initiation of various diseases, including oral cancer [18, 19]. Subsequently, after cancer development, bacteria can colonize a subset of tumors, in particular those along the digestive tract [20–22]. We and others have demonstrated that opportunistic pathogenic bacteria, such as *Fusobacterium nucleatum*, are enriched in tumors and can have a pro-tumorigenic influence on the tumor-immune microenvironment and clinical outcomes in several cancer types, including HNSCC [23–32]. However, it remains unknown why some tumors accumulate more *F. nucleatum* than other tumors within the type of same cancer.

Here, we quantified the abundance of tumor-associated bacteria, or oncobacteria, defined as total abundance of bacterial taxa significantly elevated in tumor tissue compared to adjacent normal tissue from our prior publication [24] in HNSCC tumors. We then analyzed how abundance of oncobacteria varies across clinicopathological variables in HNSCC. We find that the abundance of oncobacteria was not associated with clinical tumor stage, tumor size, or patient characteristics, but that it was decreased in HPV+ tumors. Moreover, we find that high oncobacteria abundance corresponds with poor prognosis in HPV+ tumors. We validated these observations in an internal cohort and demonstrate that these observations can be generalized to other virus-associated cancers. Finally, we establish in vitro that *F. nucleatum* preferentially inhibits the growth of HPV + HNSCC cell lines compared to HPV-negative cell lines. Together, these results indicate that tumor-intrinsic phenotypes can influence the abundance of intratumoral bacteria.

## Materials and methods

### Mammalian cell lines and culture

All cells were culture in a humidified incubator at 37 °C and 5% CO_2_, verified mycoplasma-free by the Cleveland Clinic Cell Services’ Cell and Media Production Core (CC Media Core), and routinely STR tested. No antibiotics were used during routine cell culture or experiments. FaDu (ATCC HTB-43) and UPCI:SCC152 (ATCC CRL_3240) cells were culture in EMEM (CC Media Core) supplemented with 10% FBS (Hyclone). OQ01 (provided by Dr. Lung-Ji Chang), VU-147T (provided by Dr. Timothy Chan), SCC-9 (ATCC CRL-1629), and SCC-25 (ATCC CRL-1628) cells were culture in DMEM/F12 (CC Media Core) supplemented with 10% FBS (Hyclone). PCI-15B (provide by Dr. Robert Ferris) cells were cultured in DMEM (Media Core) supplemented with 10% FBS (Hyclone). UM-SCC-47 (Sigma-Aldrich SCC071) cells were cultured in DMEM (CC Media Core) supplemented with 10% FBS (Hyclone) and Non-Essential Amino Acids (CC Media Core).

### Bacterial strains and culture

*Fusobacterium nucleatum* subsp. *nucleatum* (ATCC 23726) were grown under anaerobic conditions in TSABYEP medium consisting of Tryptic Soy Broth (Millipore) supplemented with 5 g/L Yeast Extract (BioBasic) and 10 g/L BactoPeptone (BioWorld). Bacteria were streaked on TSABYEP agar plates prepared using the same media with the addition of agar (Himedia) at 15 g/L.

### Bacterial co-culture and in vitro viability analysis

FaDu, SCC-152, OQ01, VU-147T, SCC-9, SCC-25, PCI-15B, and SCC-47 cells were seeded to flat bottom clear 96-well plates one day before co-culture. *Fusobacterium nucleatum* were re-suspended in pre-reduced mammalian cell culture media and added at a titrated multiplicity of infection (MOI) from 1000 to 15.6. Bacteria-free pre-reduced media was added to control wells. Mammalian cells and bacteria were incubated under standard anaerobic conditions for three hours, then returned to standard mammalian cell culture conditions for three days. After co-culture cells were washed with complete mammalian cell culture media two times, followed by two washes with PBS (CC Media Core). Cells were then fixed using 10% neutral buffered formalin (Millipore) for 10 min, then washed twice with PBS. Plates were then stained with Hoechst 34580 (Invitrogen) at 1:5000 for ten minutes. Plates were then imaged on the Cytation 5 Imaging System (Agilent). Relative cell number was calculated as the nuclear area in the test condition divided by the mean nuclear area in the media control, multiplied by one hundred. Experiments were replicated in biological triplicate.

### Analysis of TCGA samples

All data was obtained from the TCGA pan-cancer atlas release. Additional quantification of microbial content was obtained from Dohlman et al. [33] using PathSeq (47). Oncobacterium abundance was defined as reads of the only 3 bacterial taxa we previously found to be enriched in tumor tissue compared to adjacent normal tissue [24], that is p.*Fusobacteria*, c.*Clostridia*, and s.*Actinomyces israelii*, per million mapped reads. For analysis of HNSCC, we used bacterial reads derived from whole exome sequencing. Comparison of bacterial relative abundances was restricted to samples with more than 10 reads. Due to lower abundance of bacteria, for analysis of gastric cancer (STAD) we used bacterial reads derived from whole genome sequencing. For multivariate survival analysis via Cox proportional hazards model, Oncobacteria abundance was treated as a continuous variable along with clinical stage. For Kaplan-Meier analysis, high Oncobacteria was defined as higher than the 75^th^ percentile of adjacent normal tissue.

### Internal cohort and bacteria quantification

Representative H&E slides of tumors and paired adjacent normal tissues were identified and tissue pathology was confirmed by a head and neck pathologist. The corresponding formalin-fixed paraffin-embedded (FFPE) tissue blocks were identified. Five sections of 10 μm thickness were cut from each block for DNA isolation. The Qiagen QIAamp DNA FFPE tissue kit was utilized for isolation of DNA per protocol. 16S V4 qPCR was performed (Zymo Research). For survival analysis, samples were divided at median bacterial abundance. Patient characteristics are given in Supplementary Tables S1 and S2.

### Ethics approval and consent to participate

Experiments were performed on residual archival tissue and approved as exempt by the Institutional Review Board at University of Florida based on samples being use of residual archival samples that were completely de-identified, and not possible to attain consent for deceased patients. All experiments were performed in accordance with relevant guidelines and regulations.

### Statistics

Statistical comparisons were made using GraphPad Prism 9 (GraphPad Software) and MATLAB R2020a (Mathworks). Parametric tests were utilized due to non-normal data distributions. Variations were equivalent between groups. A comparison of two groups was made using a rank-sum test. Comparison of more than two groups was made using a Kruskal–Wallis test. All tests were two-sided. Adjustments for multiple comparisons were made with Benjamini Hochberg procedure. Comparisons between two continuous variables were made using Spearman correlation coefficient. Survival was assessed either by Cox proportional hazards model or log-rank test. Further specific details on sample sizes and statistical tests utilized are given within corresponding figure legends.

## Results

### Oncobacteria are less abundant in HPV + HNSCC than in HPV-negative HNSCC

**Fig. 1:**
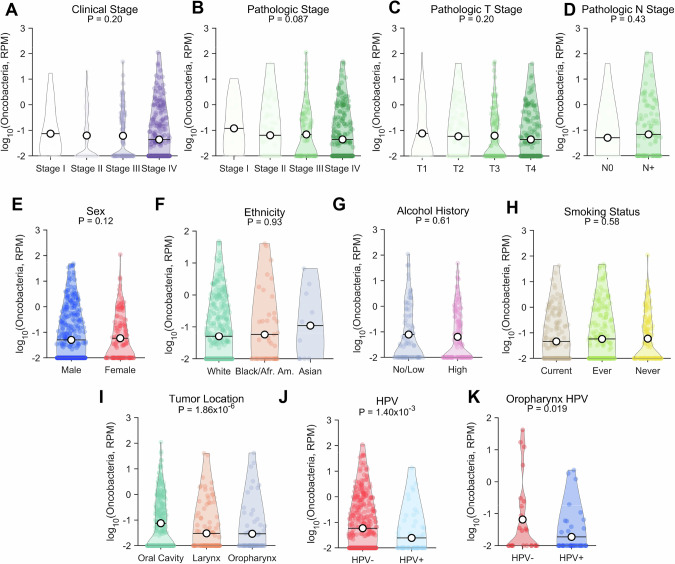
Oncobacteria are less abundant in HPV + HNSCC than in HPV-negative HNSCC. Abundance of oncobacteria, defined as reads per million (RPM), in tumors from patients with HNSCC stratified by **A** clinical stage, **B** pathologic stage, **C** pathologic T stage, or **D** pathologic node status. *n* = 511. Kruskal–Wallis test. Abundance of oncobacteria in tumors from patients with HNSCC stratified by **E** sex, **F** ethnicity, **G** alcohol history, **H** smoking status, **I** tumor location, or **J** HPV status. *n* = 511. **F**, **H**, **I** Kruskal–Wallis or **E**, **G**, **J** Mann–Whitney test. **K** Abundance of oncobacteria in tumors from patients with HNSCC of the oropharynx stratified by HPV status. *n* = 73. Rank-sum test.

To understand why some tumors accumulate more oncobacteria than others, we analyzed next-generation sequencing data in The Cancer Genome Atlas (TCGA) [33]. As recent studies have expressed concerns about analysis of microbial content from bulk sequencing, we wanted to ensure we focused our analysis on bacteria confirmed to be relevant within the intratumoral microbiome. To that end, we defined oncobacteria as the only three bacterial taxa enriched in tumors compared to adjacent normal tissues from our prior study in head and neck cancer patient surgical tissues, that is, *Fusobacteria*, *Clostridia*, and *Actinomyces* [24]. Using whole exome sequencing (WES) across HNSCC tumors, we found no differences associated with clinical stage (Fig. 1A) or pathological stage (Fig. 1B). As our oncobacteria taxa are all anaerobic bacteria, we hypothesized that tumor size and associated hypoxia may contribute to oncobacteria abundance. However, we found that tumor size, as indicated by pathological T stage (Fig. 1C), was not associated with oncobacteria abundance. We further found no association between oncobacteria abundance and pathologic node positivity (Fig. 1D). Furthermore, no differences were associated with patient biological sex (Fig. 1E) or ethnicity (Fig. 1F), nor risk factors for HNSCC including alcohol usage (Fig. 1G) and smoking (Fig. 1H). When comparing tumors based on anatomical origin location, we found oncobacteria significantly (*P* = 1.86 × 10^−6^) varied by subsite, observing highest levels in oral cavity tumors and lowest in oropharynx tumors, with intermediate values for larynx tumors (Fig. 1I). As HPV+ are predominately from the oropharynx, we next compared the abundance of oncobacteria in HPV+ and HPV-negative tumors, finding significantly more oncobacteria in HPV-negative tumors (*P* = 1.4 × 10^−3^, Fig. 1J). When restricting the comparison to tumors within the oropharynx, we found that HPV-negative oropharynx tumors still exhibited significantly more oncobacteria than HPV+ oropharynx tumors (*P* = 1.9 × 10^−2^, Fig. 1K). Analysis of relative abundance of oncobacteria either combined or individually showed no difference based HPV status (Supplementary Fig. S1).

### Oncobacteria is associated with worse prognosis in HPV+ oropharynx cancers

**Fig. 2:**
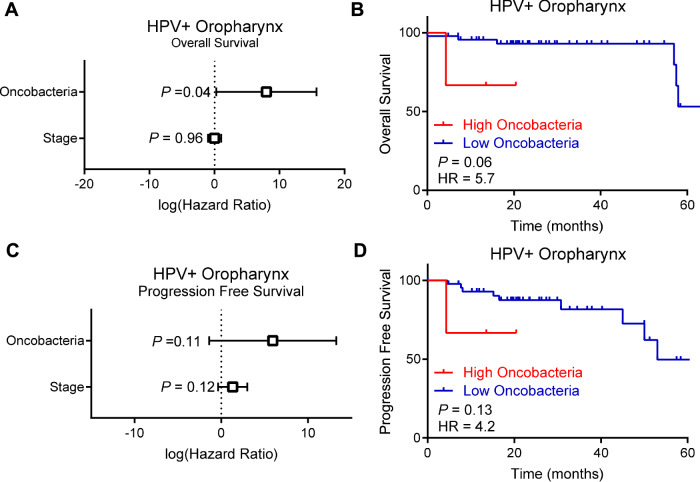
Oncobacteria are associated with worse prognosis in HPV+ oropharynx cancers. **A** Multivariate Cox proportional hazard ratios model for ability of tumor stage and oncobacteria to predict overall survival in HPV+ oropharynx samples, taking oncobacteria as a continuous variable. Error bars indicate 95% confidence interval. *n* = 49. **B** Overall survival of HPV + OPSCC patients stratified into oncobacteria high and low. Log-rank test. *n* = 49. **C** Multivariate Cox proportional hazard ratios model for ability of tumor stage and Oncobacteria to predict progression free survival in HPV+ oropharynx samples, taking Oncobacteria as a continuous variable. Error bars indicate 95% confidence interval. *n* = 49. **D** Progression free survival of HPV + OPSCC patients stratified into Oncobacteria high and low. Log-rank test. *n* = 49.

HPV-negative tumors are known to have a worse prognosis than HPV+ tumors [34], and we found that HPV-negative tumors also exhibited a higher abundance of oncobacteria. Based on this, we hypothesized that a high oncobacteria abundance may be associated with worse prognosis in HPV+ tumors. Using a Cox proportional hazards model to control for tumor stage, we found that the abundance of oncobacteria as significantly associated with worse overall survival in HPV+ oropharynx tumors (Fig. 2A). Dividing the tumors into high and low abundance for univariate analysis likewise showed a trend towards worse prognosis in HPV+ oropharynx tumors (Fig. 2B). Similar trends were observed with progression free survival using both multivariate (Fig. 2C) and univariate (Fig. 2D) analyses. Statistical power of these analyses is restricted due to the low number of recurrences in HPV+ oropharynx tumors.

### Association between oncobacteria, HPV status, and patient outcomes validates across cohorts and generalizes to other cancer types

**Fig. 3:**
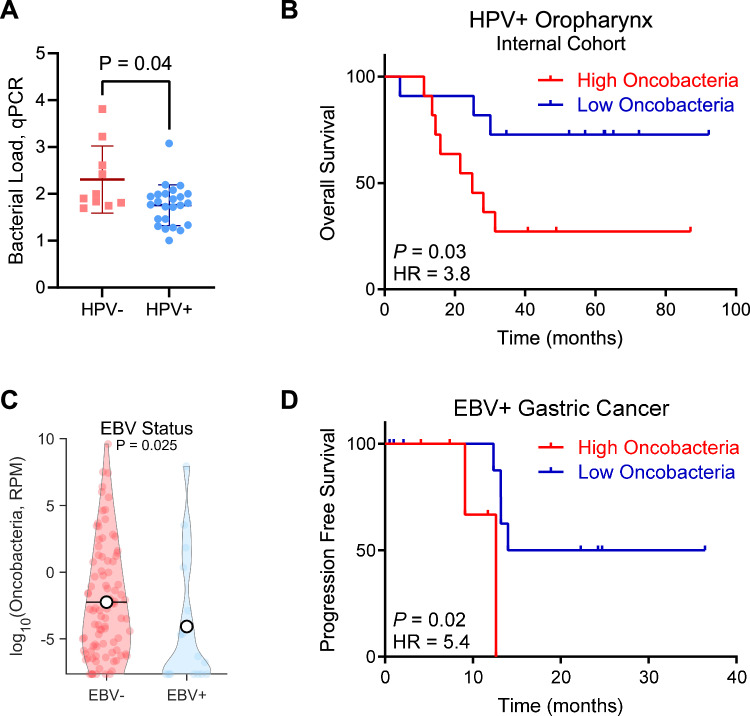
Orthogonal validation of oncobacteria associations in internal cohort and EBV-related gastric cancer. **A** Bacterial load in oropharynx tumors from internal cohort as determined by qPCR, stratified by HPV status. *n* = 34. Rank-sum test. **B** Overall survival in patients with HPV+ oropharynx tumors from (**A**), stratified by bacteria abundance. *n* = 22. Log-rank test. **C** Abundance of Oncobacteria in tumors from patients with gastric cancer stratified by EBV status. *n* = 108. Rank-sum test. **D** Progression free survival in patients with EBV+ gastric tumors from (**D**), stratified by Oncobacteria abundance. *n* = 16. Log-rank test.

To validate our observations using orthogonal quantification approaches and independent samples, we generated an internal cohort of 34 patients with HPV+ and HPV-negative oropharyngeal squamous cell carcinoma (patient characteristics given in Supplementary Table S1). Furthermore, we enriched this cohort for recurrent HPV+ tumors to overcome limitations from low recurrence rates in unselected HPV+ tumors, such as those used in the prior TCGA analysis (Fig. 2). Within this cohort, we found that HPV-negative oropharyngeal tumors exhibited significantly less tumor-associated bacteria than their HPV+ counterparts (*P* = 0.01, Fig. 3A), mirroring our original observation. Furthermore, within the HPV+ tumors we found that high bacteria abundance was associated with worse overall survival (Fig. 3B). Likewise, no differences in sex, smoking status, or stage were noted between high and low oncobacteria HPV+ oropharynx tumors (Supplementary Table S2).

To evaluate if this association between virally mediated tumors and bacteria abundance is generalizable across cancers, we next analyzed gastric cancer based on positivity for Epstein-Bar virus (EBV). We observed that EBV-negative gastric tumors exhibited a higher abundance of oncobacteria than EBV+ tumors (Fig. 3C), and that high oncobacteria was associated with worse prognosis within EBV+ gastric cancers (*P* = 0.03, Fig. 3D). Together, these results validate our observations through orthogonal approaches in oropharyngeal HNSCCs and demonstrate that the association may generalize to other cancer types.

### In vitro characterization of bacterial effects on tumor cells is HPV-dependent

**Fig. 4:**
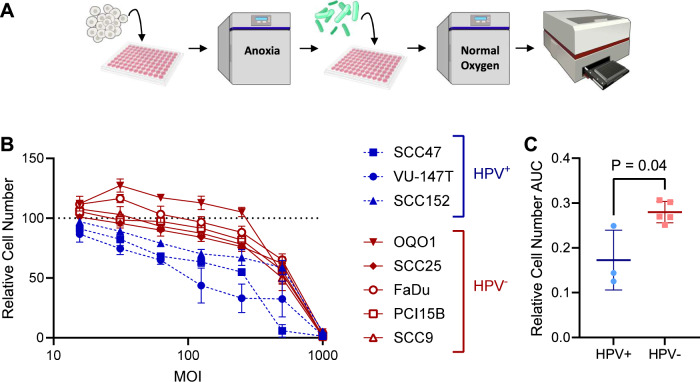
*Fusobacterium* effect on tumor cell viability is HPV-dependent. **A** Schematic of HNSCC tumor cell line and *Fusobacterium* infection experiment. *Fusobacterium nucleatum* were seeded unto the mammalian cells under anaerobic conditions, held in anoxia for 3 h and then transferred to a cell culture incubator for the duration of the experiment. Plates were washed and fixed, prior to staining with Hoechst and imaging. For detailed steps see *Materials and Methods*. Schematics adapted from BioIcons and NIH BIOART. **B** Relative cell number of mammalian HNSCC tumor cell lines infected with *Fusobacterium nucleatum* for 3 days. MOI range starting from 500 bacteria per mammalian cell, titrated 1:1. Run in biological triplicate. **C** Area under the relative cell number curve (AUC) of HNSCC tumor cell lines after 3 days infection with *Fusobacteria nucleatum*, stratified by HPV status. AUC for each cell line determined from average values shown in (**B**). HPV+, *n* = 3; HPV-negative, *n* = 5. Rank-sum test.

To determine if there are intrinsic tumor cell features associated with this altered abundance of oncobacteria in HPV+ and HPV-negative tumors, we next sought to evaluate the viability of HPV+ and HPV-negative tumors co-cultured with oncobacteria in vitro. For these studies, we selected to use *Fusobacteria* for our model bacteria, specifically *Fusobacterium nucleatum* (*Fn*). *Fusobacteria* make up the largest fraction of observed oncobacteria and are widely reported to be enriched in various cancers [23, 24, 26, 28, 28–31]. As shown in Fig. 4A, to quantify the effects of *Fn* on tumor cell lines, tumor cells were infected with varying amounts of *Fn* for 3 h under anoxic conditions and then moved to standard culture conditions for 3 days. After 3 days, cells were fixed, nuclei stained with DAPI, and subjected to high-throughput imaging to quantify relative cell number. Quantitative image analysis was selected to enable accurate quantification of tumor cells specifically without interference from *Fn* present in the culture.

We assessed the impact Fn co-culture on five HPV-negative and three HPV + HNSCC cell lines. Across all multiplicity of infection (MOI) values analyzed, HPV+ cell lines exhibited worse viability than HPV-negative cell lines (Fig. 4B). Notably, *Fn* was able to promote the proliferation of both OQ01 and FaDu HPV-negative cells at lower MOIs. Quantifying the overall impact of viability as area under the viability curve, we observed significantly lower viability for HPV+ cell lines compared to HPV-negative cell lines (Fig. 4C). The increased ability of HPV-negative HNSCC cell lines to co-exist in presence of *Fn* compared to HPV+ cell lines could suggest that tumor cell intrinsic features may contribute to the different levels of oncobacteria invasion into HNSCC tumors.

## Discussion

The prevalence of HNSCC incidence and mortality are rising globally. HPV + OPSCC has surpassed the prevalence HPV+ cervical cancers [10], and is projected to continue increasing over the next twenty years [35]. HPV+ tumors typically exhibit better prognosis than HPV-negative tumors, although approximately 30% of patients with HPV + OPSCC experience disease progression and metastasis [34]. A critical feature restricting therapeutic efficacy in HNSCC is the immunosuppressive tumor microenvironment [36]. We previously found that tumor-associated bacteria can promote an immunosuppressive microenvironment [24], and recent research has demonstrated a role for intratumoral bacteria in chemo-resistance via a variety of mechanisms [37, 38]. However, what determines variations in bacterial abundances remained unknown. Here, we demonstrate that abundance of tumor-associated bacteria differs by HPV status, with HPV-negative patient tumors harboring more oncobacteria and HPV-negative cell lines showing better viability when co-cultured with *Fn* oncobacteria than their HPV+ counterparts. Notably, the few HPV+ tumors with high oncobacteria exhibited a significantly worse prognosis. Taken together with the observation that HPV-negative tumors have worse prognosis than HPV+ tumors and also harbor more oncobacteria, these findings suggest intrinsic features of more aggressive tumors facilitate oncobacteria colonization to exacerbate treatment difficulties.

The molecular mechanisms underlying HPV-negative tumors being able to harbor more bacteria than HPV+ tumors warrant further investigation. HPV is a double-stranded circular-DNA virus of approximately 8,000 base pairs that infects the dividing basal keratinocytes of the oropharynx [39], with HPV-16 the most commonly associated with oropharyngeal squamous cell carcinomas and cervical cancers. The majority of patients clear HPV infections within two years, though some persistent infections occur leading to the development of premalignant lesions and invasive carcinoma [40]. The HPV genome consists of several open reading frames encoding the viral machinery; named according to their timing of expression during the viral life cycle, including ‘E’ (early) or ‘L’ (late) [41]. The major oncoproteins of HPV are E6 and E7 which are known to inhibit the tumor suppressors p53 [E6] and pRb [E7], whereas p53 is preferentially inactivated via mutation in HPV-negative tumors [42]. HPV oncoproteins also greatly impact multiple other pathways including; PI3K, AKT, Wnt, Notch and epithelial-mesenchymal transition (EMT) [43]. These oncoproteins have also been shown to facilitate tumor progression through immune evasion, by downregulation of the interferon response [43] and proliferation, through binding to Octamer binding transcription factor-4 (Oct4) [44]. Genetically, HPV+ tumors have been shown to be enriched in alterations in *TRAF3*, *E2F1*, and *FGFR3* [42]. Conversely, HPV-negative tumors further harbor more alterations in other cell cycle genes (*CCND1*/*CDKN2A*), growth factor receptors (*EGFR*/*FGFR1*/*IGF1R*), and oxidative stress genes (*NFE2L2*/*KEAP1*) [42]. Moreover, HPV+ tumors are known to be more immunogenic than HPV-negative tumors, with increased levels of adaptive immune cells (46). Taken together with the aforementioned role of bacteria in promoting an immunosuppressive microenvironment [24], future studies further delineating a potential causal role of bacteria in remodeling the tumor-immune microenvironment are warranted.

These molecular differences between HPV+ and HPV-negative tumors could provide initial molecular candidates to explain observed differences in ability to proliferate in the presence of oncobacteria.

In conclusion, this work demonstrates that tumor molecular characteristics may contribute to the varying levels of oncobacteria observed in HNSCC tumors and that high levels of oncobacteria may identify HPV+ tumors with worse prognosis. These findings may be critical to our understanding and treatment of HPV+ tumors which are rapidly increasing in prevalence.

## Supplementary information

Supplemental Material

## Data Availability

Data from TCGA samples were downloaded from the GDC data commons (https://portal.gdc.cancer.gov/) or from Dohlman et al. [33].
