# Supplementary material for: HPV status impacts oncobacteria abundance and prognostic relevance in head and neck squamous cell carcinoma
Source: Oncogene. 2025 Jun 10;44(26):2217–23. doi: 10.1038/s41388-025-03463-4 (PMC12183080; doi:10.1038/s41388-025-03463-4)
Supplement: Supplementary file 1 — Supplemental Material [file 41388_2025_3463_MOESM1_ESM.pdf]

Supplementary Materials for:

HPV status impacts oncobacteria abundance and prognostic relevance in head and neck squamous cell carcinoma

Travis D. Kerr[1,2]*, Natalie L. Silver[1,3]*, Radhika Duggal[1], Jin Dai[1], Hannah Simmons[1], Subha Signh[1,4], Akeesha Shah[5], Kristianna Fredenburg[6], Apollo R. Stacy[7], Daniel J. McGrail[1#]

[1]Center for Immunotherapy and Precision Immuno-Oncology, Lerner Research Institute, Cleveland Clinic, Cleveland, OH, USA
[2]Department of Biochemistry and Molecular Biology, University of Miami, Miller School of Medicine, Miami, FL, USA
[3]Head and Neck Institute, Cleveland Clinic, Cleveland, OH, USA
[4]Department of Molecular Medicine, Cleveland Clinic Lerner College of Medicine, Case Western Reserve University, Cleveland, OH, USA
[5]Pathology and Laboratory Medicine Institute, Cleveland Clinic, Cleveland, OH, USA
[6]Department of Pathology, University of Florida College of Medicine, Gainesville, Florida, USA
[7]Department of Cardiovascular and Metabolic Sciences, Lerner Research Institute, Cleveland Clinic, Cleveland, OH, USA

*These authors contributed equally to this work.

Correspondence to Daniel J. McGrail (mcgraid@ccf.org)

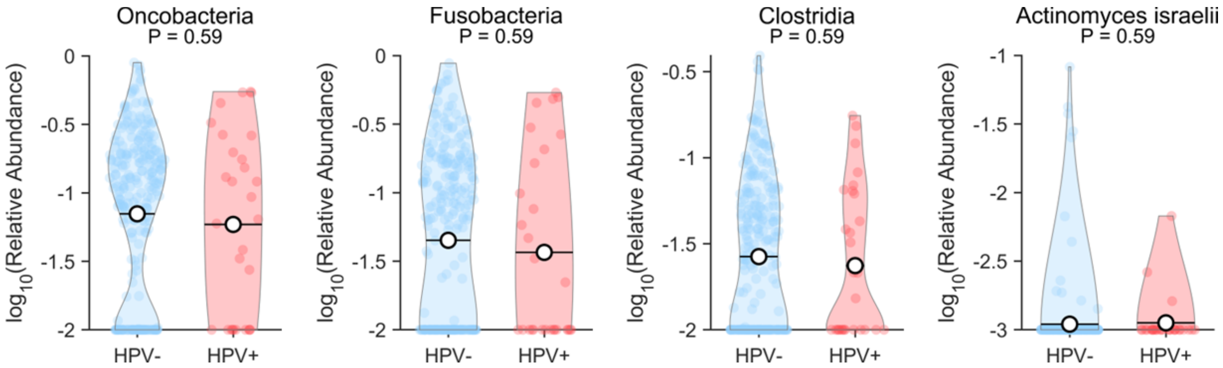

Figure S1. Oncobacteria relative abundance by HPV status.
Relative abundance of either all oncobacteria or individual taxa contributing to oncobacteria using whole exome sequencing data. Rank-sum test with Benjamini Hochberg correction for multiple comparisons. n = 294.

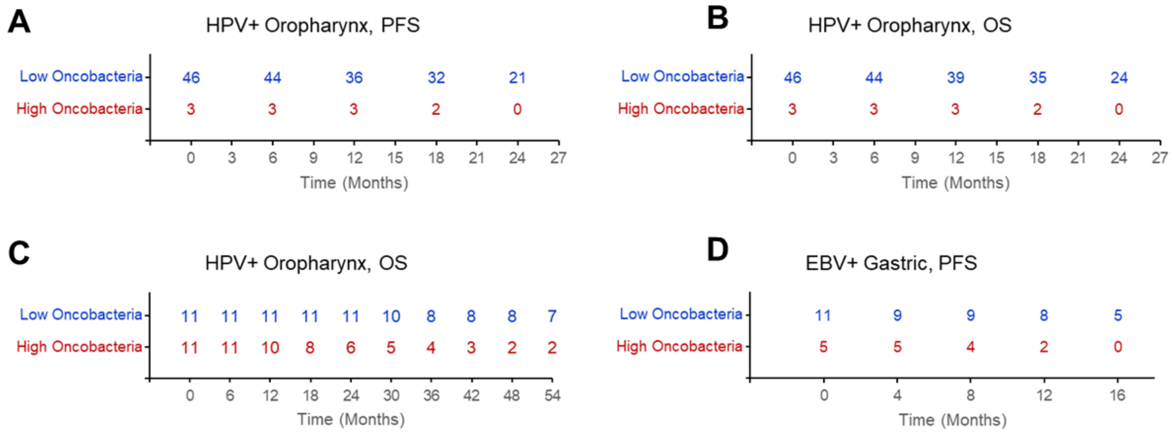

Figure S2. Risk tables for PFS/OS curves.
(A) Risk table associated with HPV+ oropharynx TCGA PFS data form Figure 2B
(B) Risk table associated with HPV+ oropharynx TCGA OS data form Figure 2D
(C) Risk table associated with HPV+ oropharynx internal data from Figure 3B
(D) Risk table associated with EBV+ gastric TCGA PFS data form Figure 3D

Table S1. Patient characteristics for entire oropharynx internal cohort by HPV status.

Characteristics	HPV- (N = 10)	HPV+ (N = 23)
**Sex (P = 0.0018)**		
Male	5 (50.0%)	22 (95.7%)
Female	5 (50.0 %)	1 (4.3%)
**Age (P = 0.98)**		
Mean (St.Dev.)	58.1 (8.8)	58.7 (8.6)
**Smoking status (P = 0.06)**		
Never Smoker	1 (10.0%)	10 (43.5%)
Ever Smoker	9 (90.0%)	13 (56.5%)
**Subsite (P = 0.79)**		
Tongue	3 (30.0%)	8 (34.8%)
Tonsil	7 (70.0%)	15 (65.2%)
**T Stage (P = 0.85)**		
1	1 (10.0%)	4 (17.4%)
2	3 (30.0%)	7 (30.4%)
3	2 (20.0%)	6 (26.1%)
4	4 (40.0%)	6 (26.1%)
**N Stage (P = 0.27)**		
0	5 (50.0%)	4 (17.4%)
1	1 (10.0%)	4 (17.4%)
2	4 (40.0%)	14 (60.1%)
3	0 (0.0%)	1 (4.3%)
**M Stage (P = 1.0)**		
0	10 (100%)	23 (100%)
**Overall AJCC8 Stage (P = 4.2×10^{-4})**		
1	0 (0%)	5 (21.7%)
2	3 (30.0%)	9 (39.1%)
3	1 (10.0%)	9 (39.1%)
4	6 (60.0%)	2 (8.7%)

Table S2. Patient characteristics for HPV+ oropharynx internal cohort by bacteria abundance.

Characteristics	Low (N = 12)	High (N = 11)
**Sex (P = 0.29)**		
Male	12 (100%)	10 (90.9%)
Female	0 (0 %)	1 (9.1%)
**Age (P = 0.58)**		
Mean (St.Dev.)	57.8 (9.9)	59.6 (7.2)
**Smoking status (P = 0.85)**		
Never Smoker	5 (41.7%)	5 (54.5%)
Ever Smoker	7 (58.3%)	6 (45.5%)
**Subsite (P = 0.79)**		
Tongue	3 (30.0%)	8 (34.8%)
Tonsil	7 (70.0%)	15 (65.2%)
**T Stage (P = 0.14)**		
1	2 (16.7%)	2 (18.2%)
2	4 (33.3%)	3 (27.3%)
3	5 (41.7%)	1 (9.1%)
4	1 (8.3%)	5 (45.4%)
**N Stage (P = 0.25)**		
0	3 (25.0%)	1 (9.1%)
1	3 (25.0%)	1 (9.1%)
2	5 (41.7%)	9 (81.8%)
3	1 (8.3%)	0 (0%)
**M Stage (P = 1.0)**		
0	12 (100%)	11 (100%)
**Overall AJCC8 Stage (P = 0.37)**		
1	4 (33.3%)	1 (9.1%)
2	4 (33.3%)	5 (45.4%)
3	4 (33.3%)	5 (45.4%)
4	0 (0%)	0 (0%)
